# Enhanced Nutrients Removal Using Reeds Straw as Carbon Source in a Laboratory Scale Constructed Wetland

**DOI:** 10.3390/ijerph15061081

**Published:** 2018-05-27

**Authors:** Tong Wang, Haiyan Wang, Yang Chang, Zhaosheng Chu, Yaqian Zhao, Ranbin Liu

**Affiliations:** 1 School of Civil Engineering, Chang’an University, Xi’an 710061, China; wangt@chd.edu.cn (T.W.); cy1100@126.com (Y.C.); 2 State Key Laboratory of Environmental Criteria and Risk Assessment, Chinese Research Academy of Environmental Sciences, No. 8 Da Yang Fang, Anwai, Chaoyang District, Beijing 100012, China; chuzs@craes.org.cn; 3 Research Center for Water Pollution Control Technology, Chinese Research Academy of Environmental Sciences, No. 8 Da Yang Fang, Anwai, Chaoyang District, Beijing 100012, China; 4 National Engineering Laboratory for Lake Pollution Control and Ecological Restoration, Chinese Research Academy of Environmental Sciences, Beijing 100012, China; 5 State Key Laboratory of Eco-Hydraulic Engineering in Arid Area, Xi’an University of Technology, Xi’an 710048, China; 6 UCD Dooge Centre for Water Resources Research, School of Civil Engineering, Newstead Building, University College Dublin, Belfield, Dublin 4, Ireland; liu.ranbin@ucdconnect.ie

**Keywords:** C/N ratio, constructed wetland, denitrification, nutrient removal, reeds straw

## Abstract

The low carbon/nitrogen (C/N) ratio and high nitrate content characteristics of agricultural runoff restricted the nitrogen removal in constructed wetlands (CWs). To resolve such problems, the economically- and easily-obtained *Phragmites Australis* (reeds) litters were applied and packed in the surface layer of a surface flow CW as external carbon sources. The results demonstrated that the introduction of the reeds straw increased the C concentration as a result of their decomposition during the CW operation, which will help the denitrification in the ensuing operation of an entire 148 days. The total nitrogen (TN) and Chemical Oxygen Demand (COD) () in the effluent reached the peak level of 63.2 mg/L and 83 mg/L at the fourth and the second day, respectively. Subsequently, the pollutants in the CW that were filled with straw decreased rapidly and achieved a stable removal after 13 days of operation. Moreover, the present study showed that the N removal efficiency increased with the increase of the hydraulic retention time (HRT). Under the HRT of four days, the CW presented 74.1 ± 6%, 87.4 ± 6% and 56.0 ± 6% removal for TN, NO_3_^-^, and TP, respectively.

## 1. Introduction

Non-point source pollution (NSP), including stormwater runoff, acid mining drainage, and agricultural runoff, has become a very severe issue all over the world and it is even worse in China [1]. Agricultural NSP has been identified as one of the major NSPs in China [2]. It is easy to understand as fertilizers or pesticides are adopted in the farmland and the fields to increase the productivity. Along with the rough management of fertilizer utilization, larger amount of nutrients (nitrogen [N] and phosphorus [P]) in the soil are carried through runoff to the streams and/or reservoirs, and it is even infiltrated into the groundwater. The excess leakage of nutrients into the surface water has overwhelmed the self-purification capacity of the waterbodies and destroyed the balance of input and depletion. Therefore, the accumulated N and/or P in the streams or reservoirs has induced severe eutrophication. The bloom of the phytoplankton/algae has consequently deteriorated the water resource quality and posed a threat to other aquatic lives [3].

There are a number of approaches for the agricultural operations in order to reduce the nutrients content in the runoff. The key point are rooted in the collaboration between a wide range of people and organizations across an entire watershed. Specifically, despite the nutrient management of applying fertilizers in the proper amount and at the right time, adopting buffers between farmland and waterbodies is an essential solution so as to absorb or filter out nutrients before they reach the surface water. The buffers could be natural- or manmade-projects, depending on the pollution intensity of the runoff.

Constructed wetland (CW) is an artificially intensified biofilm-like process and has been widely applied in the treatment of domestic and municipal wastewater, and even in landfill leachate [4]. Moreover, it is also an effective process for runoff treatment because of its easy design and operation, as well as its cost-effectiveness. Generally, the treatment performance of the CW is highly associated with the properties of the selected matrix and wetland plants, and is good for organic matter (biochemical oxygen demand (BOD_5_) and chemical oxygen demand (COD)) removal [5]. For achieving effective P removal, some special matrix could be adopted, such as zeolite, clay, drinking water residue, and iron/calcium ore etc., which possess a robust P adsorption ability [6]. However, for N removal, denitrification is the dominating process for TN depletion, under the circumstance where sufficient electron donor (organic carbon source) is available [7]. The problem is that agricultural runoff usually has a low C/N ratio, such as C/N = 0.8. Therefore, it is difficult to achieve satisfying TN removal in CW systems [8]. Although the addition of external liquid carbon sources, such as glucose, methanol, ethanol, starch, and sodium acetate, is a common method to alleviate the carbon deprivation and to improve the efficiency of biological denitrification in CWs [9], it is not sustainable, as the liquid carbon source needs to be added continuously, which is in high-cost. Therefore, seeking an easy operation, popularization, and low cost carbon source to enhance N removal are highly desirable in CW research and development.

From the literature, rice husk, litter, woodchips, sawdust, cotton, maize cobs, seaweed, and bark have all been reported in the last decade as external solid carbon sources to enhance denitrification [10]. However, in CWs, plant litter, as one abundant carbon-contained material, has gained increasing attention for being used as a carbon resource, because of its cost-effectiveness. During the decomposition of plants, a large amount of organic compounds could be released, which could be available for denitrification [10]. Chen et al. [11] reported that the *Typha latifolia* litter addition could greatly improve nitrate removal in subsurface-batch CWs through the continuous input of organic carbon. Li et al. [12] demonstrated the use of corn straw, after pretreatment of soaking the corn straw in an alkaline solution for treating agricultural drainage water with a low C/N ratio in a horizontal subsurface flow CW. The average removal efficiency of TN and TP in tested CW increased by 37.2% and 30.5%, respectively, under the hydraulic retention time (HRT) of three days. Reeds are one of the most widely distributed wetland plant genera worldwide, with high productivity. Therefore, it has been considered as a common carbon-containing source in the CWs, in order to balance the C/N of the agricultural runoff. However, few studies have been reported on the treatment of agricultural drainage water using reeds straw as carbon source in CWs. This forms the basis of the current study.

The aim of the present study was to resolve the lack of carbon source and other issues in low C/N ratio runoff, with reed litters in a laboratory scale surface flow CW. During the 148 days of study, the C release patterns from the added reeds straw in the CW were monitored, while N removal behavior was explored. Moreover, the HRT was studied and optimized from an engineering scope. It is expected that the current study could provide useful information and a showcase on low C/N wastewater treatment when using surface flow CW.

## 2. Materials and Methods

### 2.1. CW Design and Reeds Straw Preparation

**Figure 1:**
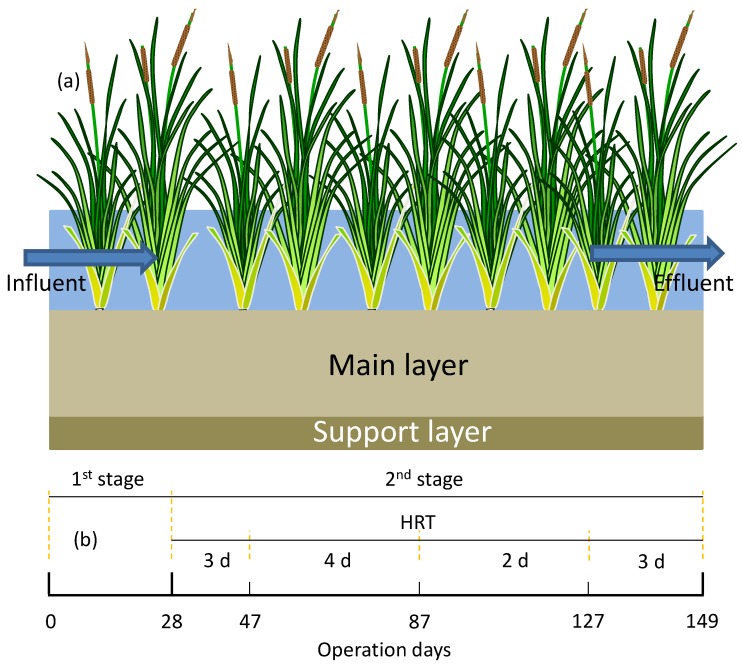
Schematic diagram of the reactor and the operation timeline. HRT—hydraulic retention time.

Two CW reactors (Figure 1a) that were made of stainless steel were built in a greenhouse in a countryside near Erhai, Yunnan Province, China, with a dimension of 120 × 40 × 80 (L × W × H) cm. Both of the CWs were applied to the top soil (size of 5–10 mm) of a local farmland as a wetland substrate. The substrate in the reactors comprised of two layers, the main substrate layer (30 cm) covered on the supporting layer of 10 cm of coarse soil. A mixture of soil and air-dried reeds straw as the main substrate in CW I were employed. CW II was filled with only soil as main substrate. The reeds straw were prepared from the harvested mature reeds in the Erhai riparian natural wetland. After exposure outside to dry, the reeds were chopped into 1~2 cm bars and then mixed with the soil [12]. The total mass of the reed bars was about 9.2 kg with a specific filling quantity of 19.2 kg/m^2^. In CW I, sand with a 1 cm was overlaid on the top of the main substrate so as to prevent the reeds straw from floating. The porosity of CW I was 0.13–0.14 with a working volume of 140 L. Young reeds and cattails were planted on the surface of two CWs with a density of 9 plants/m^2^.

### 2.2. Water Preparation and Operation Scheme

The raw water that was used as influent in the present study was artificial water that was based on the typical local agricultural runoff (Table 1) [13]. The water was composed of (in mg/L): 17.5 ± 1.2 TN, 16.4 ± 0.8 NO_3_^−^-N, 0.5 ± 0.3 NH_4_^+^-N, 1.12 ± 0.1 TP, and 14 ± 0.8 COD.

In order to initiate the biological treatment, the CWs were inoculated with a diluted activated sludge and were operated under static mode (data was not presented) for one week before shifting to a continuous operation, during which the reactors were operated in a free water mode [14]. In the start-up stage (static mode), extra carbon source was supplied so as to promote the establishment of microorganisms. In the first stage (0–28 days) of the continuous operation, an HRT of three days was adopted in order to evaluate the release of the nutrients from the straw. Then, from days 28 to 149 (2nd stage), the impact of the HRT (two days, three days, and four days) [12] on the pollutant removal was studied by changing the influent flow rate, as depicted in Figure 1b. The influent was introduced into the CWs using a peristaltic pump, and the effluent overflowed out of the wetland from three drainage ports. The free water depth of each CW was 25 cm. The effluent was regularly sampled once every two days and the TN, TP, NO_3_^−^, NH_3_, and COD were analyzed using a HACH DR/2400 spectrophotometer, according to its standard operating procedures [15].

## 3. Results and Discussion

### 3.1. C and N Release Pattern in the CWs of the First Stage

**Figure 2:**
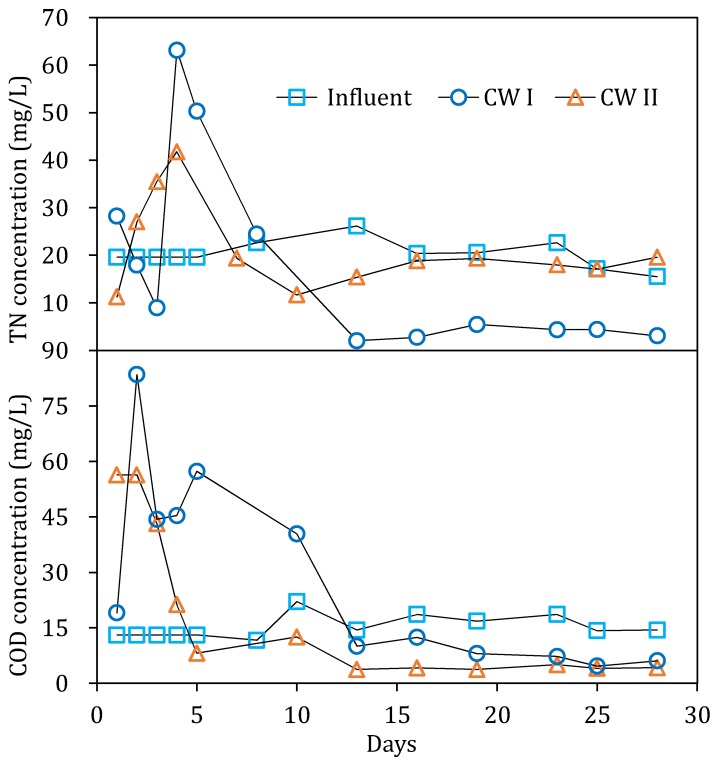
TN and COD content in the influent and effluent of two CWs in the first stage.

Previous studies [16] had indicated that about 20.1% of the reeds straw could have been decomposed in the first 21 days. Therefore, it was very important to reveal the release pattern of the organic and the nitrogen from the reeds straw in the CWs, as the release pattern was obviously of great significance in guiding the engineering practice. The results of the first stage regarding the concentration of TN and COD in the effluent of each CW are depicted in Figure 2.

The content of TN in the influent of both CWs remained around 20 ± 3 mg/L. However, the TN in the effluent increased at the early stage (1–8 days). It was easy to understand that the degradation and/or decomposition of the nitrogen-contained compounds in the soils and the reeds straws (in CW I) contributed to the increase of the TN. The exclusive matrix of the reeds straw in the CW I obviously induced a higher TN release, as depicted in Figure 2. Both CWs quickly reached a TN peak concentration on the fourth day, which was 63.2 mg/L and 41.8 mg/L in CW I and II, respectively. Then, the residual TN in the effluent of the two CWs decreased gradually before becoming stable. Obviously, the decrease of the TN content in both of the CWs was attributed to the overwhelming N depletion rate, as a result of the denitrification compared with the N release rate. After the CWs were operated for 13 days, the TN concentrations in the effluent of both of the CWs were lower than the influent. At this point, the effluent TN concentration in CW I was 3.6 ± 0.5 mg/L, much lower than CW II of 18.5 ± 1 mg/L. During this stage, the plant had not yet grown completely, so the nitrogen removal contribution from the plant was negligible. Genssner [17] pointed out that the rise of the TN concentration was related to microbial solidification under the reed decomposition process. It was reasonable to believe that the main reasons for the elevated levels of N in the CWs were as follows: (1) Non-nitrogen compounds decomposed rapidly, (2) microbial growth and biomass increased, and (3) microbial had curing effect on the surrounding media nitrogen.

The COD release pattern was a little bit different from the TN. As depicted in Figure 2, the COD in CW II reached a peak value of 54 mg/L on the first day. As a result of the decomposition and dissolution of the reed residue, the COD in CW I reached its peak concentration on the second day, that is, 83 mg/L. This result was consistent with previous studies [16]. After the peak concentration, the COD in the effluent began to decrease because of the increasing formation of the microbial biomass. On the 13th day, the residual COD in the effluent was almost stable, with a mild decline. At the end of this stage, the level even decreased to 4 mg/L.

Overall, the TN and COD in the effluent of both of the CWs experienced a sharp increase at the early stage before achieving a net pollutant removal. After about two weeks, the residual pollutants, especially the TN in the effluent of CW I, had obviously decreased and were lower than that in CW II, which had been as a result of the establishment of microorganisms in the reactor. Overall, the release of pollutants from the reeds straw was unlikely to be avoided at the early stage or before the introduction of bacteria. It would be a big concern for extrapolating the lab-scale reactor to field practice. Thus, appropriate measures should be considered so as to address the early effluent from the CW that was filled with reeds straw, for example, the pretreatment of the reeds straw before filling it into the reactor, or collecting and recirculating the early effluent into the reactor.

### 3.2. Nitrogen Removal Performance in the Second Stage

**Figure 3:**
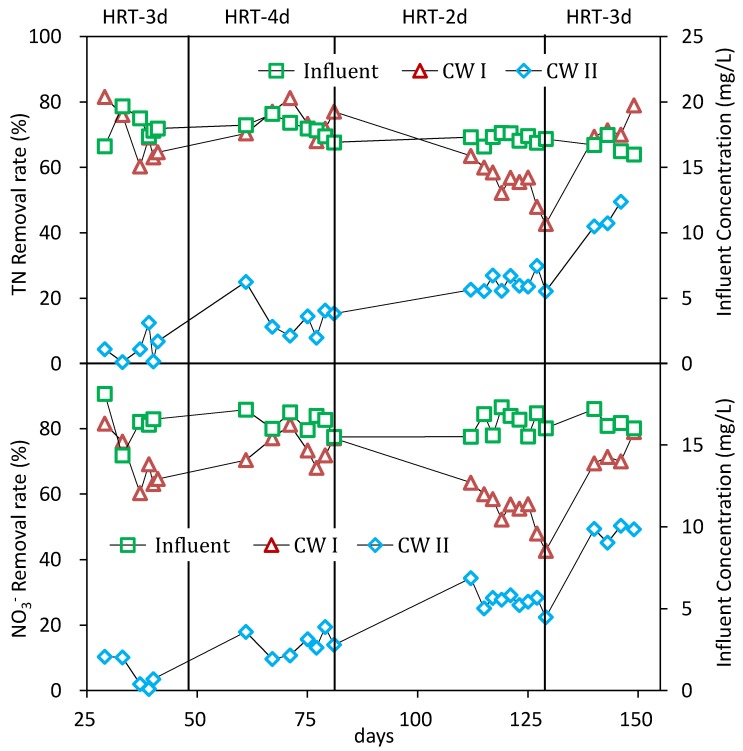
The influent concentration and removal efficiency of TN (**upper)** and NO_3_^−^ (**bottom**) in the second stage.

Generally, many pathways contributed to the nitrogen removal in CW, including volatilization, ammonification, nitrification/denitrification, plant uptake, and matrix adsorption [18]. In the case of possessing adequate carbon sources, denitrification was the main contribution. In order to confirm the optimal HRT for TN removal, a series of HRT (two, three, and four days) were selected for confirmation in the second stage and the results are depicted in Figure 3. It showed that the TN and NO_3_^−^ removal efficiencies in CW I were 64.3 ± 5%, 74.1 ± 6%, and 53.0 ± 5%, and 81.0 ± 9%, 87.4 ± 6%, and 62.1 ± 2% under an HRT of three, four, and two days, respectively. Correspondingly, the efficiencies in CW II were 14.4 ± 3%, 14.4 ± 3%, and 24.0 ± 3%, and 14.4 ± 4%, 14.4 ± 4%, and 26.8 ± 4%, respectively. Obviously, the removal efficiencies of TN and NO_3_^−^ had increased with the prolonged HRT. Moreover, the CW II without reeds straw filled had achieved a poor performance compared with the CW I, regardless of the HRT condition. Obviously, the TN removal efficiency was restrained by the insufficient carbon source that was needed to fuel the denitrification. By the contrast, CW I benefited from the reeds straw that was filled inside, which supplied the carbon source.

**Figure 4:**
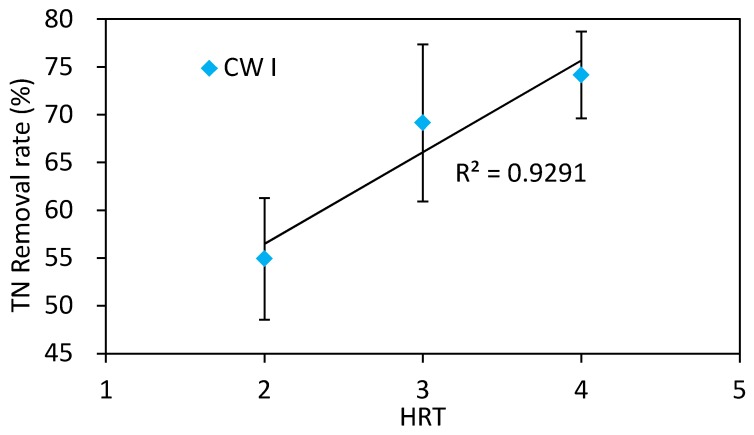
Correlation of the TN removal efficiency and the HRT.

In order to make the results more cogent, the HRT and the nitrogen removal efficiency were correlated by the analysis of regression, as depicted in Figure 4. The R^2^ was as high as 0.93, which demonstrated a significantly positive correlation in CW II. It meant, for CW II, that the HRT of four days was the optimal condition for achieving satisfying TN removal. Based on the trend in Figure 4, the TN removal performance would be improved further by increasing the HRT for longer. However, this would sacrifice the high flow rate or it would lessen the land footprint that was needed to build the CW. Thus, proper HRT should be selected in the full-scale application.

It was also noteworthy that the nitrogen removal schemes in the two CWs along with the operation time showed different results. For CW II, the nitrogen removal efficiency gradually increased. During days 130–139, the SFW was reset to run under the HRT of three days. The results showed that the removal efficiency of TN and NO_3_^−^ had increased to 44.1 ± 6% and 48.5 ± 5%, respectively. The possible reasons for this included the following: (a) the plants’ contribution to enhanced nitrogen removal with the operation time; (b) the growth of wetland plants which could release some sugars, alcohols, and acids into the CW; and (c) the decay of the roots and leaves, which provided the carbon source for denitrification. This was consistent with the previous study [19]. However, after 129 days, the removal efficiency of NO_3_^−^ in CW I had decreased from 81 ± 9% to 73.9 ± 7%. At this time, although the plants’ contribution to denitrification had enhanced, the carbon source from the reeds straw had decreased. On the other hand, the TN removal efficiency of CW I had increased from 64.3 ± 5% to 71.8 ± 8%.

### 3.3. Other Forms of N Accumulation

A previous study [19] had alleged that the decomposition of aquatic plants and incomplete denitrification could have induced the accumulation of NH_3_ and NO_2_^-^. The problem was that the accumulation of NH_3_ and NO_2_^−^ had some toxic effects on the growth of plants and microorganisms in the system. In order to verify this concern, the NH_3_ and NO_2_^−^ were monitored and the results are summarized in Table 1. After stable operation of CW II and CW I, the NO_2_^-^ and NH_3_ content in the effluent were accumulated at a small level. Although their concentrations were not high enough to destroy the stability of the wetland system, special attention needed to be paid to it in order to prevent the ecological risk.

### 3.4. TP Removal Efficiency in 2nd Stage

**Figure 5:**
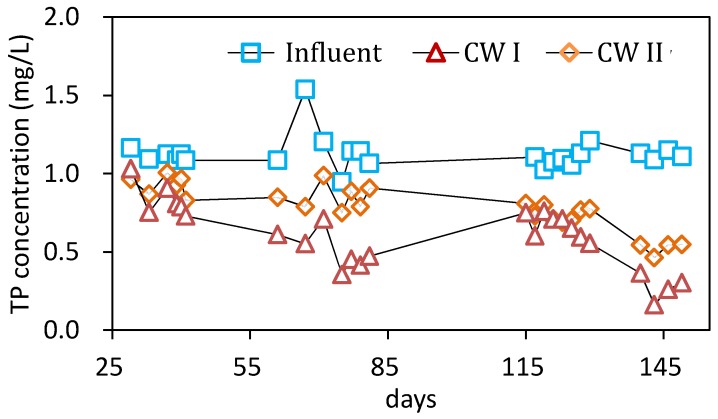
Influent and effluent concentration of TP in the second stage.

Figure 5 depictes the P removal performance under different HRT, with the same influent P content of 1.12 ±0.6 mg/L. The results showed that the CW II achieved 17.3 ± 7%, 21.0 ± 3%, and 33.0 ± 2% of P removal under the HRT of three, four, and two days, respectively. By the contrast, the P removal efficiencies in the CW I for three, four, and two days were 27.0 ± 7%, 56.0 ± 6%, and 38.0 ± 7%, respectively. Under each HRT, the removal efficiency in the CW I was obviously better than that in CW II. For the CW I reactor, the HRT of four days was the best for the P removal, which was inconsistent with the nitrogen removal. Moreover, after 130 days of operation, the removal efficiency of TP had increased to 64 ± 10% in CW I and 53 ± 4% in CW II.

A previous study [20] showed that the wetland bottom accumulation, which had co-precipitation with the plant residues by the adsorption of P compounds, played an important role in removing P. CW I with vast reeds straw, to help increasing matrix organic matter, also had a positive impact in strengthening the matrix P co-precipitation. In addition, the results showed that when the COD concentration was less than 500 mg/L, increasing the COD concentration could promote the TP removal.

### 3.5. Perspective

Although further study will be needed to explore the mechanisms behind the COD and N release and the interactions with denitrification for TN removal, this study has provided a good showcase of the reeds straw in a potential application, in practice. Particularly, the surface flow of the CW was regarded as having had less efficiency in CWs for wastewater treatment [21]. However, it was still in popular use in some cases, in order to avoid wetland substrate clogging, which was the biggest problem in CW practice. Therefore, the results from the current study provided confidence that the reeds straw addition could be a good strategy and technical solution for low C/N wastewater treatment, by a considerable enhancement of TN and TP removal of 59.7% and 35.0%, respectively, at HRT of four days. Although it was questionable to compare this enhancement with other cases of different materials being added and the varied operation condition, as well as different CW system configuration, the enhancement was comparable from the limited literature, 37.2% for TN and 30.5% for TP [12]. Chen et al. [11] had concluded that denitrification contributed to 54–94% N removal in CWs. There was no doubt that reeds straw addition, as an external carbon source, could have accelerated the denitrification process. Obviously, more work was desirable, in order to quantify this process in detail.

## 4. Conclusions

In the present study, the role of reeds straw in promoting pollutants (N and P) removal in a CW was verified for treating agricultural runoff. The results demonstrated the benefits of involving reeds straw as a carbon-release material in enhancing the pollutants removal. The main findings can be summarized as follows.

(1) The existence of reeds straw in the CW induced more C and N release during the initial stage. The TN (63.5 mg/L) and COD (83 mg/L) reached its peak release on the third day and the second day, respectively. After 13 days, the CW achieved a stable pollutant net removal.

(2) The nitrogen removal efficiency with reeds straw as a carbon source was obviously improved, which was reflected by the control CW. Moreover, the nitrogen removal increased with the increasing HRT. In the present study, four days was the optimal HRT, with an average of 74.1% TN removal being achieved, which was 14.4% in the CW without reeds straw addition.

(3) The addition of the reeds straw also promoted P removal by a possible increase of adsorption and complexation sites. A net increase of a TP removal of 35% was achieved from 21% in the absence of reeds straw, and 56% with the reeds straw addition.

## Figures and Tables

**Table 1:** The NH_3_ and NO_2_^−^ accumulation under different hydraulic retention time (HRT).

Reactor	HRT	Influent (mg/L)		Effluent (mg/L)	
		NH_3_	NO_2_^−^	NH_3_	NO_2_^−^
CW II	2	0.4 ± 0.2	0.01 ± 0.01	0.1 ± 0.1	0.06 ± 0.02
	3	0.5 ± 0.2	0.01 ± 0.01	0.2 ± 0.1	0.86 ± 0.4
	4	0.4 ± 0.2	0.01 ± 0.01	0.1 ± 0.1	0.04 ± 0.02
CW I	2	0.4 ± 0.2	0.01 ± 0.01	0.3 ± 0.1	0.17 ± 0.02
	3	0.5 ± 0.2	0.01 ± 0.01	1.0 ± 0.3	1.99 ± 0.5
	4	0.4 ± 0.2	0.01 ± 0.01	0.8 ± 0.3	0.69 ± 0.3
